# Supplementary material for: Investigation of Clinical Features and Association between Vascular Endothelial Injury Markers and Cytomegalovirus Infection Associated with Thrombotic Microangiopathy in Patients with Anti-Neutrophil Cytoplasmic Antibody (ANCA)-Associated Vasculitis: Case-Based Research
Source: Int J Mol Sci. 2024 Jan 9;25(2):812. doi: 10.3390/ijms25020812 (PMC10815804; doi:10.3390/ijms25020812)
Supplement: Supplementary file 1 [file ijms-25-00812-s001.zip › Supple_TableS1_TMA_AAV_v0.1_20231204.pdf]

Table S1. Laboratory data at the admission

Urinalysis		(Reference range)
Proteinuria	3+	(-)
Hematuria	3+	(-)
Red blood cells (/HPF)	100>	(-)
Red blood cell casts (/WF)	50-99	(-)
Protein/Creatinine ratio (g/gCr)	3.42	(<0.3)
Complete blood count		
White blood cells (/mm^2)	48,280	(3,500–8,000)
Neutrophil (%)	97	(38.6–72.3)
Lymphocyte (%)	3	(19.6–51.0)
Monocyte (%)	0	(2.7–7.9)
Eosinophil (%)	0	(0.6–10.1)
Red blood cells ($\times 10^4/\mu L$)	288	(435–555)
Hemoglobin (g/dL)	6.8	(11.0–14.5)
Platelet ($\times 10^4/\mu L$)	30.3	(13.0–35.0)
Prothrombin time (sec)	14.3	(10.0–43.0)
Activated partial thromboplastin time (sec)	29.0	(23.0–38.0)
Fibrinogen (mg/dL)	675.0	(180.0–350.0)
D-dimer (mg/dL)	36.4	(0.0–1.0)
Soluble thrombomodulin (IU/mL)	44.1	(<7.0)
Pentraxin 3 (ng/mL)	55.3	(0.73–5.49)
Serum chemistry		
Total protein (g/dL)	5.7	(6.7–8.3)
Albumin (g/dL)	1.1	(3.8–5.3)
Urea nitrogen (mg/dL)	78.0	(8.0–22.0)
Creatinine (mg/dL)	3.59	(0.40–0.80)
Uric acid (mg/dL)	9.8	(2.6–7.0)
Total cholesterol (mg/dL)	104	(142–219)
Aspartate aminotransferase (IU/L)	13	(10–30)
Alanine aminotransferase (IU/L)	5	(5–40)
Lactate dehydrogenase (IU/L)	266	(110–220)
Sodium (mEq/L)	139	(135–147)
Potassium (mEq/L)	3.8	(3.5–5.0)
Chloride (mEq/L)	107	(98–108)
Calcium (mEq/L)	6.8	(8.5–10.2)
Phosphorus (mEq/L)	7.1	(2.5–4.5)
Glucose (mg/dL)	75	(75–110)
Hemoglobin A1c (%)	5.4	(4.6–6.2)
C-reactive protein (mg/dL)	25.2	(<0.1)
Immunoglobulin G (mg/dL)	2187	(861–1747)
Immunoglobulin A (mg/dL)	367	(93–393)
Immunoglobulin M (mg/dL)	128	(33–183)
Complement 3 (mg/dL)	90.0	(70–150)
Complement 4 (mg/dL)	20.3	(14–40)
50% hemolytic complement activity (IU/mL)	47.5	(30–50)
Antinuclear antibody	1280	(-)
Anti-double-stranded deoxyribonucleic acid antibody (IU/mL)	9.5	(0.0–12.0)
Anti SSA antibody	(-)	(-)
Anti-SSB antibody	(-)	(-)
Anti Smith antibody (IU/mL)	0.1	(<10.0)
Myeloperoxidase-antineutrophil cytoplasmic antibody (IU/mL)	71.6	(0.0–3.5)
Protease3- antineutrophil cytoplasmic antibody (IU/mL)	<1.0	(0.0–3.5)
Anti-glomerular basement membrane antibody (IU/mL)	<1.0	(<3.0)
Hepatitis B virus surface antigen (IU/mL)	0.001	(0.00–0.004)
Hepatitis C virus antibody (IU/mL)	0.1	(0.0–0.9)
Human immunodeficiency virus antibody (IU/mL)	0.1	(0.0–0.9)
Cytomegalovirus immunoglobulin G antibody (AU/mL)	202.0	(0.0–15.0)
