# Supplementary material for: Investigation of Clinical Features and Association between Vascular Endothelial Injury Markers and Cytomegalovirus Infection Associated with Thrombotic Microangiopathy in Patients with Anti-Neutrophil Cytoplasmic Antibody (ANCA)-Associated Vasculitis: Case-Based Research
Source: Int J Mol Sci. 2024 Jan 9;25(2):812. doi: 10.3390/ijms25020812 (PMC10815804; doi:10.3390/ijms25020812)
Supplement: Supplementary file 1 [file ijms-25-00812-s001.zip › Supple_TableS2_TMA_AAV_v0.2_20231226.pdf]

Table S2. Laboratory data at the emergence of TMA

Complete blood count		(Reference range)
White blood cells (/mm^2)	51,580	(3,500–8,000)
Neutrophil (%)	97	(38.6–72.3)
Lymphocyte (%)	2	(19.6–51.0)
Monocyte (%)	1	(2.7–7.9)
Eosinophil (%)	0	(0.6–10.1)
Red blood cells (×10^4/μL)	327	(435–555)
Hemoglobin (g/dL)	7.9	(11.0–14.5)
Platelet (×10^4/μL)	1.5	(13.0–35.0)
Shistocyte	(+)	(-)
Prothrombin time (sec)	14.5	(10.0–43.0)
Activated partial thromboplastin time (sec)	32.1	(23.0–38.0)
Fibrinogen (mg/dL)	232.00	(180.0–350.0)
D-dimer (mg/dL)	30.1	(0.0–1.0)
Thrombin-antithrombin III complex (μg/L)	5.5	(<3.0)
Antithrombin III (%)	71.9	(80.0–120.0)
Disseminated intravascular coagulation score on Japanese diagnostic criteria	5	(0–6)
Serum chemistry		
Total protein (g/dL)	4.4	(6.7–8.3)
Albumin (g/dL)	1.6	(3.8–5.3)
Urea nitrogen (mg/dL)	105	(8.0–22.0)
Creatinine (mg/dL)	3.36	(0.40–0.80)
Uric acid (mg/dL)	9.7	(2.6–7.0)
Aspartate aminotransferase (IU/L)	22	(10–30)
Alanine aminotransferase (IU/L)	7	(5–40)
Lactate dehydrogenase (IU/L)	446	(110–220)
Sodium (mEq/L)	137	(135–147)
Potassium (mEq/L)	4.5	(3.5–5.0)
Chloride (mEq/L)	104	(98–108)
C-reactive protein (mg/dL)	3.2	(<0.1)
Haptoglobin (mg/dL)	<5	(25–176)
Myeloperoxidase-antineutrophil cytoplasmic antibody (IU/mL)	71.6	(0.0–3.5)
A disintegrin-like and metalloproteinase with thrombospondin type 1 motif 13 activity (%)	19.5	(>10.0)
A disintegrin-like and metalloproteinase with thrombospondin type 1 motif 13 antibodies (IU/mL)	0.0	(<0.5)
Direct coombs	(-)	(-)
Indirect coombs	(-)	(-)
Heparin-induced thrombocytopenia antibody (IU/mL)	0.6	(0.0–0.9)
Cryoglobulin	(-)	(-)
Anti-cardiolipin immunoglobulin G antibody (IU/mL)	1.0	(<12.3)
Anti-beta2-glycoprotein 1 antibody (IU/mL)	<1.2	(<3.5)
