# Supplementary material for: Investigation of Clinical Features and Association between Vascular Endothelial Injury Markers and Cytomegalovirus Infection Associated with Thrombotic Microangiopathy in Patients with Anti-Neutrophil Cytoplasmic Antibody (ANCA)-Associated Vasculitis: Case-Based Research
Source: Int J Mol Sci. 2024 Jan 9;25(2):812. doi: 10.3390/ijms25020812 (PMC10815804; doi:10.3390/ijms25020812)
Supplement: Supplementary file 1 [file ijms-25-00812-s001.zip › Supple_TableS3_CMV_test25case_v0.1_20231121.pdf]

Supplementary Table S3. Main clinical characteristics and three vascular endothelial injury markers in AAV patients who developed CMV viremia and those who did not.

	Analyzed patients (N=25)	CMV viremia (+) (N=15)	CMV viremia (-) (N=10)	*P*-value
Age	72 (12)	71 (14)	75 (7)	0.37
Male (n,%)	12 (48.0)	7 (46.7)	5 (50.0)	1.00
Body mass index (kg/m^2)	22.1 (3.3)	23.1 (3.4)	20.6 (2.8)	0.06
Total protein (g/dL)	6.2 (0.8)	6.1 (0.8)	6.4 (0.8)	0.28
Albumin (g/dL)	2.6 (0.6)	2.5 (0.6)	2.9 (0.7)	0.21
eGFR (ml/min/1.73m^2)	10.9 [7.2, 14.7]	9.8 [7.7, 12.7]	12.9 [7.6, 23.5]	0.32
C reactive protein (mg/dL)	4.25 [0.54, 8.72]	6.10 [0.79, 8.82]	1.83 [0.50, 7.04]	0.44
White blood cell count (/μL)	6880 [5810, 11780]	10670 [7220, 14440]	5400 [4843, 6590]	0.006
Hemoglobin (g/dL)	9.3 (1.6)	9.5 (1.7)	9.0 (1.6)	0.52
Platelet count (×10^4/μL)	30.4 (13.8)	28.1 (14.6)	33.8 (12.4)	0.32
Total cholesterol (mg/dL)	174 (36)	170 (35)	179 (38)	0.54
Triglyceride (mg/dL)	120 [104, 166]	124 [107, 167]	114 [99, 139]	0.42
Serum sulfatides (nmol/mL)	5.32 (2.16)	4.94 (2.21)	5.89 (2.07)	0.29
Lysosulfatides-d18:2 (nmol/mL)	1.54 (0.75)	1.43 (0.73)	1.73 (0.79)	0.34
Lysosulfatides-d18:1 (nmol/mL)	3.35 (1.28)	3.11 (1.36)	3.71 (1.13)	0.26
Lysosulfatides-d18:0 (nmol/mL)	0.07 (0.11)	0.09 (0.10)	0.04 (0.11)	0.35
Lysosulfatides-t18:0 (nmol/mL)	0.35 (0.21)	0.32 (0.22)	0.41 (0.19)	0.29
sTM (U/mL)	50.1 [39.4, 55.3]	54.2 [44.7, 63.8]	39.0 [33.2, 45.4]	0.005
Pentraxin 3 (ng/mL)	28.4 [6.0, 53.2]	15.2 [6.3, 55.3]	6.8 [6.1, 39.5]	0.44

Continuous variables exhibiting a normal distribution are presented as means and standard deviations, while those exhibiting a non-normal distribution are presented as medians and interquartile ranges. Categorical variables are presented as numbers (n) and percentages (%). Comparisons of continuous variables between two groups (CMV viremia (+) and (-)) were conducted by Student's t-test or Mann–Whitney U test for variables with a normal or nonnormal distribution, respectively. The comparison of categorical variables was performed using the Chi-squared test. A p-value < 0.05 was considered statistically significant.

AAV; anti-neutrophil cytoplasmic antibody-associated vasculitis, CMV; cytomegalovirus, eGFR; estimated glomerular filtration rate, sTM; soluble thrombomodulin.
