# Supplementary figures and images for: Investigation of Clinical Features and Association between Vascular Endothelial Injury Markers and Cytomegalovirus Infection Associated with Thrombotic Microangiopathy in Patients with Anti-Neutrophil Cytoplasmic Antibody (ANCA)-Associated Vasculitis: Case-Based Research
Source: Int J Mol Sci. 2024 Jan 9;25(2):812. doi: 10.3390/ijms25020812 (PMC10815804; doi:10.3390/ijms25020812)

## Slide 1
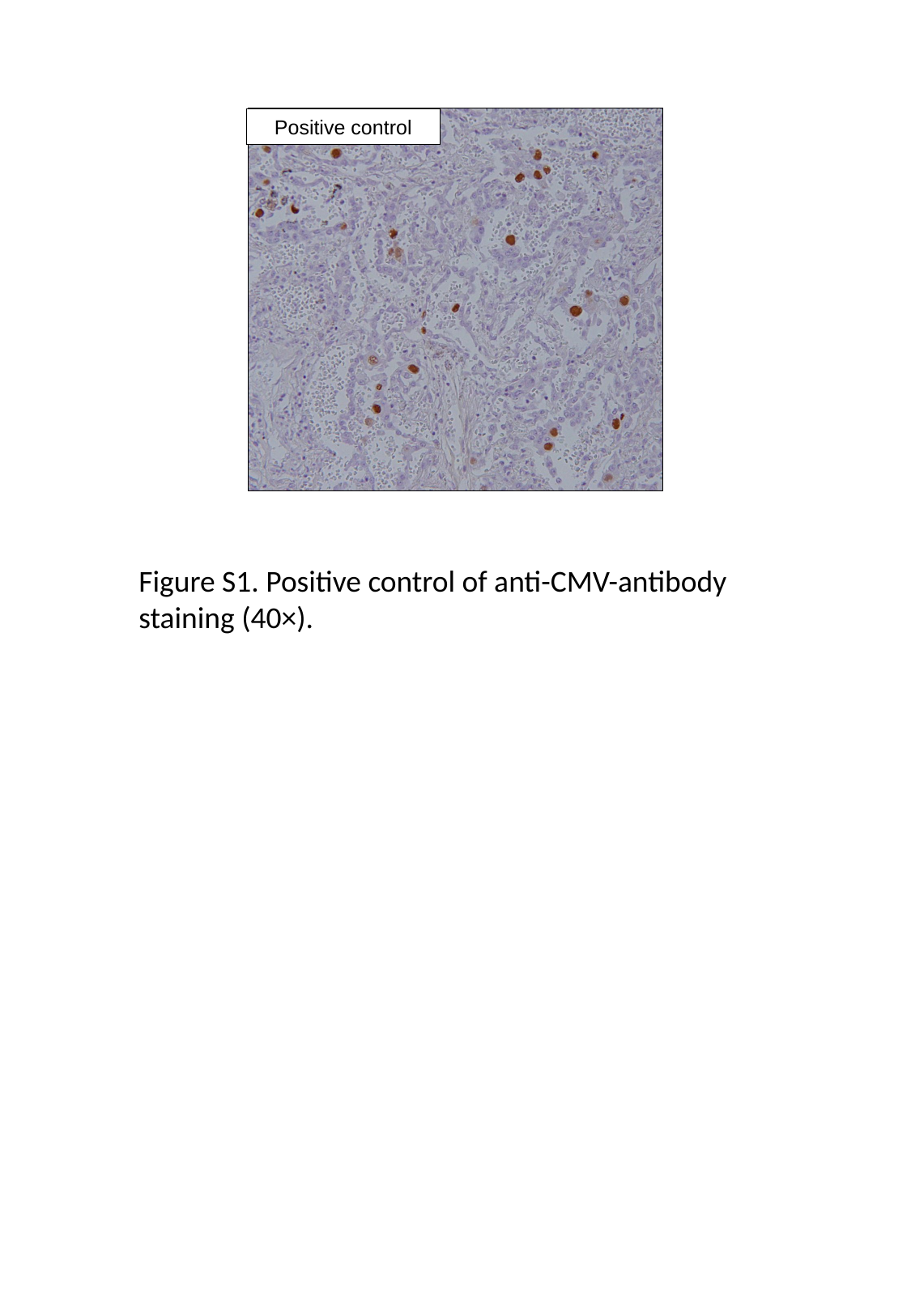

Positive control
Figure S1. Positive control of anti-CMV-antibody staining (40×).

Supplement: Supplementary file 1 [file ijms-25-00812-s001.zip › Supple_figS1_TMA_AAV_positive control.pptx]
